# The Human Gut Colonizer *Blastocystis* Respires Using Complex II and Alternative Oxidase to Buffer Transient Oxygen Fluctuations in the Gut

**DOI:** 10.3389/fcimb.2018.00371

**Published:** 2018-10-22

**Authors:** Anastasios D. Tsaousis, Karleigh A. Hamblin, Catherine R. Elliott, Luke Young, Alicia Rosell-Hidalgo, Campbell W. Gourlay, Anthony L. Moore, Mark van der Giezen

**Affiliations:** ^1^RAPID Group, Laboratory of Molecular & Evolutionary Parasitology, School of Biosciences, University of Kent, Canterbury, United Kingdom; ^2^School of Biological and Chemical Sciences, Queen Mary University of London, London, United Kingdom; ^3^Biosciences, University of Exeter, Exeter, United Kingdom; ^4^Department of Biochemistry and Biomedicine, School of Life Sciences, University of Sussex, Brighton, United Kingdom; ^5^Kent Fungal Group, School of Biosciences, University of Kent, Canterbury, United Kingdom

**Keywords:** *Blastocsytis*, Complex II, alternative oxidase, gut microbiome, oxygen tolerance, mitochondria

## Abstract

*Blastocystis* is the most common eukaryotic microbe in the human gut. It is linked to irritable bowel syndrome (IBS), but its role in disease has been contested considering its widespread nature. This organism is well-adapted to its anoxic niche and lacks typical eukaryotic features, such as a cytochrome-driven mitochondrial electron transport. Although generally considered a strict or obligate anaerobe, its genome encodes an alternative oxidase. Alternative oxidases are energetically wasteful enzymes as they are non-protonmotive and energy is liberated in heat, but they are considered to be involved in oxidative stress protective mechanisms. Our results demonstrate that the *Blastocystis* cells themselves respire oxygen via this alternative oxidase thereby casting doubt on its strict anaerobic nature. Inhibition experiments using alternative oxidase and Complex II specific inhibitors clearly demonstrate their role in cellular respiration. We postulate that the alternative oxidase in *Blastocystis* is used to buffer transient oxygen fluctuations in the gut and that it likely is a common colonizer of the human gut and not causally involved in IBS. Additionally the alternative oxidase could act as a protective mechanism in a dysbiotic gut and thereby explain the absence of *Blastocystis* in established IBS environments.

## Introduction

A healthy human gut is characterized by the presence of obligate anaerobic bacteria from the Firmicutes and Bacteroides phyla who are considered to play a protective role in maintaining the gut ecosystem (Donaldson et al., 2016). The establishment of this protective microbial ecosystem in the gut has its origin within the first few days/weeks after birth and even the mode of birth (cesarean or natural) can result in effects in later life (Sommer et al., 2017). The resilience of the gut ecosystem, which co-evolved with humans, is illustrated by the fact that generally, the gut flora returns to its original state after perturbations, such as antibiotic treatment or infections (Donaldson et al., 2016; Sommer et al., 2017). However, certain perturbations can result in a detrimental new equilibrium that is not beneficial to the human host. For example, an increase in facultative anaerobic *Enterobacteriaceae* is generally linked to a dysbiosis of the gut, where an increase in the luminal bioavailability of oxygen causes a shift in intestinal biodiversity (Rigottier-Gois, 2013; Byndloss et al., 2017; Rivera-Chávez et al., 2017). Recently, a mechanistic coupling between gut microbes and the presence of molecular oxygen was described by Byndloss et al. Activation of a colonocyte peroxisome proliferator-activated receptor-γ (PPARγ) results in reduction of the nitrate and oxygen concentrations in the gut thereby controlling the proliferation of facultative anaerobes (Byndloss et al., 2017). This clearly demonstrates a link between oxygen in the human intestine and dysbiosis as previously hypothesized by Rigottier-Gois (2013).

Intestinal dysbiosis has been linked to several diseases including obesity and irritable bowel diseases, such as Crohn's disease and ulcerative colitis and to irritable bowel syndrome (IBS) (Rigottier-Gois, 2013; Goulet, 2015). IBS is a common gastrointestinal disease presenting with abdominal pain, constipation, diarrhea and bloating (Enck et al., 2016). It is now generally accepted that IBS is accompanied by a changed microbial gut flora (Simrén et al., 2013) which seems adapted to higher oxygen levels in the gut (Rigottier-Gois, 2013) based on reported increases in *Enterobacteriaceae* in IBS patients (Carroll et al., 2012). Although most studies focus on bacterial taxa in IBS patients, some studies have assessed the contribution of microbial eukaryotes (Engsbro et al., 2012; Nourrisson et al., 2014; Krogsgaard et al., 2015; Nash et al., 2017). Specifically, *Blastocystis* is frequently associated with IBS, however its role in disease is contested (Clark et al., 2013; Ajjampur and Tan, 2016; Gentekaki et al., 2017; Stensvold and van der Giezen, 2018). Although it is the most common microbial eukaryote of the human gut, which can reach a prevalence of up to 100% (El Safadi et al., 2014), little is known about its virulence (Ajjampur and Tan, 2016; Ajjampur et al., 2016). This limited amount of information is compounded by the massive genetic diversity observed between isolates (Stensvold et al., 2007; Ajjampur and Tan, 2016; Gentekaki et al., 2017). Currently, *Blastocystis* is considered to be a strict anaerobe (Zierdt, 1986), which makes its role in the IBS gut even more confusing, especially considering the conflicting reports linking it to IBS (Nourrisson et al., 2014; Krogsgaard et al., 2015). *Blastocystis* received additional attention due to its unusual mitochondrion (Lantsman et al., 2008; Stechmann et al., 2008; Müller et al., 2012; Gentekaki et al., 2017). As an anaerobe, and similar to other anaerobic microbial eukaryotes, it has lost many classic features of mitochondria and performs no oxidative phosphorylation and lacks a standard mitochondrial electron transport chain (see for example van der Giezen, 2011). It has retained Complex I which supposedly maintains a proton motive force across the inner membrane and passes electrons via rhodoquinone to a fumarate reductase (Stechmann et al., 2008) which acts as an alternative Complex II (Tielens et al., 2002; Müller et al., 2012). It has also retained a mitochondrial genome (Pérez-Brocal and Clark, 2008). In addition to the canonical mitochondrial iron-sulfur cluster assembly system *Blastocystis* also has a prokaryotic SUF system that was localized in its cytosol (Tsaousis et al., 2012). Recently, it was also shown that *Blastocystis* contains part of glycolysis in its mitochondrion (Bártulos et al., 2018). Although *Blastocystis* can produce some ATP via substrate level phosphorylation using the TCA cycle enzyme succinyl-CoA synthetase (Hamblin et al., 2008) it is mainly reliant on fermentation producing lactate, acetate and probably propionate (Stechmann et al., 2008; Müller et al., 2012). The *Blastocystis* mitochondrion is predicted to contain an alternative oxidase which “should” accept electrons from Complex I and II (Stechmann et al., 2008; Standley and van der Giezen, 2012). Alternative oxidases are non-protonmotive quinol–oxygen oxidoreductases which couple the oxidation of ubiquinol to the 4-electron reduction of molecular oxygen to water (Moore and Albury, 2008). These enzymes are found in several non-related organisms. Their physiological role is not completely clear but it has been suggested to be involved in oxidative stress protective mechanisms, heat generation and to maintain tricarboxylic acid cycle turnover under high cytosolic phosphorylation potential (Moore and Albury, 2008). Alternative oxidases have been found in other parasites, such as *Cryptosporidium* (Roberts et al., 2004) and trypanosomes (Nihei et al., 2002). The trypanosome homolog is well-studied as it is considered a potential drug target due to its absence in humans (Shiba et al., 2013).

Here, we report the biochemical characterization of an alternative oxidase in *Blastocystis* and relate this to the organism's ability to cope with fluctuating oxygen concentrations in the gut and its postulated role in disease.

## Materials and methods

### Organisms and culture conditions

*Blastocystis* strain NandII cDNA was obtained from the *Blastocystis hominis* EST project (Stechmann et al., 2008). Human *Blastocystis* sp. isolate DMP/02-328 was obtained during routine screening and was grown at 36°C with a mixed bacterial flora in LYSGM with 5% adult bovine serum. Cells were grown under anoxic conditions and all culturing work performed in an anaerobic chamber (Ruskinn SCI-tive with HEPA Hypoxia station). LYSGM is a modification of TYSGM-9 in which the trypticase and yeast extract of the latter are replaced with 0.25% yeast extract (Sigma) and 0.05% neutralized liver digest (Oxoid). Subtyping of *Blastocystis* sp. DMP/02–328 indicated that this strain is subtype 4 (Stensvold et al., 2007) whereas *Blastocystis* sp. NandII is subtype 1.

*Escherichia coli* strain α select silver efficiency (Bioline) was used for cloning and heme deficient *E. coli* strain FN102 (ΔhemA (Km^R^)) (Nihei et al., 2003) was used for recombinant *Blastocystis* AOX expression.

### AOX cloning, expression and purification

The putative AOX gene was originally identified in the *Blastocystis hominis* EST project (http://amoebidia.bcm.umontreal.ca/pepdb) using BLASTn with the ESTs as queries. Full-length genes were obtained by 5′ and 3′ rapid amplification of cDNA ends using the GeneRacer Kit (Invitrogen). AOX sequences from *Blastocystis, Sauromatum guttatum*, and *Trypanosoma brucei* were aligned using ClustalW (Chenna et al., 2003) and examined.

*Blastocystis* AOX was amplified from cDNA using the forward primer 5′-aga aga *CAT ATG* TTC CCT ATC CTC TCC AGA GTC TTC-3′ and the reverse primer 5′-tct tct *GGA TCC* TTA CGC TTT CGT TGC GCC GTA CTT CG-3′ which added *Not*I and *Bam*HI restriction sites (indicated in italics), respectively. Amplification was carried out with Phusion High-Fidelity DNA polymerase (New England Biolabs) yielding amplicons of the expected size (~0.9 kb). PCR products were purified using QIAquick Gel Extraction Kit (QIAGEN), digested with *Bam*HI and *Not*I restriction digestion enzymes and cloned into pET-14b (Novagen). The pET-14b vector added a 6XHis tag to the N-terminus of AOX. The AOX pET-14b plasmid was purified using QIAprep Spin Miniprep Kit (QIAGEN), sequenced to confirm its validity (MWG) and used to transform FN102 *E. coli* cells.

FN102 membrane purification was carried out as described by Nihei et al. (2003) with minor modifications. Briefly, starter cultures of K broth with ampicillin, kanamycin and aminolevulinic acid (ALA) were inoculated and incubated at 37°C until they reached an OD_600_ of 0.1. Starter cultures were added to large scale cultures of K broth with carbenicillin until they reached an OD_600_ of 0.01. Large-scale cultures were grown at 30°C until they reached OD_600_ of 0.1, induced with 100 μM IPTG and incubated for 8 h at 30°C. Cells were harvested by centrifugation at 3,500 g for 20 min at 4°C. Harvested cells were resuspended in 30 ml of Buffer S (60 mM Tris-HCl pH 7.5, 5 mM DTT, 300 mM NaCl, 20% sucrose). Cells were broken with a sonicator and centrifuged twice at 4,000 g for 10 min at 4°C to pellet cell debris. The supernatant was layered on top of buffer G (60 mM Tris-HCl pH 7.5, 5 mM DTT, 300 mM NaCl, 40% sucrose) to create a sucrose gradient and centrifuged at 200,000 g for 1 h at 4°C. Pelleted membranes were resuspended in ~0.5 ml of buffer S.

### AOX assay

AOX activity was determined polarographically following uptake of oxygen using a Clark-type electrode (Rank Brothers, Cambridge, U.K.) using 0.1–0.5 mg *E. coli* membranes suspended in 0.4 ml air-saturated reaction medium (250 μM at 25°C) containing 50 mM Tris–HCl (pH 7.5). Data were recorded digitally using a PowerLab/4SP system (ADInstruments Pty, UK) with Chart version 3.6s software (ADInstruments).

### Western blotting

*Blastocystis* whole-cell protein lysate, from strain DMP/02-328, was separated on a 10% sodium dodecyl sulfate (SDS) polyacrylamide gel and blotted on to nitrocellulose membrane (Bio-Rad). Anti-*Sauromatum guttatum* AOX (1:1,000) was used as primary antibody followed by anti-mouse HRP conjugate (Pierce) 1:10,000 as a secondary antibody. Signal was detected using a CN/DAB Substrate Kit (Pierce). Different cell fractions were isolated following procedures previously described (Tsaousis et al., 2014). *Blastocystis* cells from NandII strain (well-grown in media for 5 days) were harvested by centrifugation at 1,200 × g for 10 min at 4°C. Cells were resuspended in Locke's solution (pH 7.4) and pelleted again at the same speed for the same duration. Cells were then broken with 40 strokes in a 10-ml Potter-Elvehjem tissue homogenizer at 4°C in isotonic buffer (200 mM sucrose, pH 7.2, 30 mM phosphate, 15 mM β-mercaptoethanol, 30 mM NaCl, 0.6 mM CaCl_2_, 0.6 mM KCl). Broken cells were diluted with isotonic buffer and then centrifuged at 700 × g for 10 min using a Sorvall RC-2B centrifuge to remove unbroken cells. The supernatant was collected and centrifuged at 5,000 × g for 20 min to pellet the large granular fraction (LGF), where MROs are found (see Lantsman et al., 2008; Tsaousis et al., 2014; Bártulos et al., 2018). The LGF was resuspended (washed) in isotonic buffer and pelleted as described above. Finally, all fractions were stored at −20°C in NuPAGE LDS sample buffer along with 10× sample reducing agent (Invitrogen). Depending on the amount of protein, 5 to 20 μl of the supernatant was analyzed using a polyacrylamide mini gel and subsequently blotted as above. Cellular fractions were analyzed using anti-*Blastocystis* hydrogenase (Stechmann et al., 2008) (1:250) and anti-*Blastocystis* SufCB (Tsaousis et al., 2012) (1:500) antisera, as controls for the mitochondrial organelle and cytoplasm, respectively. A loading control is shown in **Figure 3B**.

### Immunolocalization of AOX

*Blastocystis* cells were resuspended in 1 X phosphate buffered saline (PBS) pH 7.4 and were transferred to pretreated poly-L-lysine slides (Sigma). Slides were incubated at 4°C for 2 h and then washed for 5 min in 1X PBS. The cells on the slides were fixed with 3.7% formaldehyde/0.5% acetic acid for 15 min at 37°C. Slides were washed for 5 min in PBS/0.5% Tween-20 and then permeabilized with 0.1% Triton X-100 for 5 min. Washes were performed three times for 5 min in PBS/0.05% Triton X-100 for 5 min. Fixed cells were incubated for 30 min with a blocking solution of 5% skimmed milk powder in 1X PBS solution (w/v) and then rinsed with 0.5% milk/PBS solution for 30 min. The cells were then incubated with an anti-*S. guttatum* AOX antibody (1:100 dilution) in 1% milk/PBS solution overnight at 4°C. After three rinses in 1% milk/PBS for 10 min, the slides were incubated with a fluorescent dye-labeled (Alexa 488 green) goat secondary antibody at a dilution of 1:200. For colocalization experiments, before fixation, cells were incubated for 20 min with 200 nm of MitoTracker Red CMXRos (Molecular Probes). Cover slips were mounted with anti-fade mounting medium (Vectashield) and observed under an Olympus IX81 fluorescence microscope. Images were collected using Micromanager 1.4 software and processed with ImageJ.

### High-resolution respirometry

Oxygen consumption was measured in *E. coli* control or AOX expressing cells using a high-resolution respirometer (Oxygraph-2k; Oroboros) calibrated to 37°C in LB media and data recorded using DatLab software. Changes in the rate of oxygen consumption were measured following repeated additions of salicylhydroxamic acid (SHAM) to give a final concentrations of 1.2, 3.6, 7.0 and 9.4 mM or the appropriate volume of the carrier solvent (ethanol) as a control.

Cultured *Blastocystis* cells were collected anaerobically and gently pelleted at 800 × g for 10 min and re-suspended in sterile anoxic Locke's solution to a cell density of 1 × 10^6^ cells/ml. The oxygen consumption rate was measured using a high-resolution respirometer (Oxygraph-2k; Oroboros) calibrated to 28°C and data were recorded at 1 s intervals using DatLab software. The effects on oxygen consumption following addition of the AOX inhibitor, SHAM (Sigma), at 2.4 or 4.8 mM, or the succinate dehydrogenase inhibitor, TTFA (Sigma), at 5.4 or 11 μM as indicated. Ethanol was used as the solvent for both SHAM and TTFA whilst DMSO was used for antimycin A (1 μM) (Sigma) and octylgallate (OG) (11 μM) (Sigma).

### Protein modeling

*Blastocystis* AOX was modeled to the TAO crystal structure (PDB:5GN2) using the Swiss-model software (http://swissmodel.expasy.org/; Arnold et al., 2006; Benkert et al., 2011; Biasini et al., 2014). The protein structure of *Blastocystis* AOX was loaded into MOE software (Molecular Operating Environment, version 2016.08, Chemical Computing Group Inc., Montreal, Canada) for some preparatory steps to correct any structural issues. Hence, the QuickPrep panel was used to optimize the hydrogen bond network using the Protonate 3D algorithm and to perform an energy minimization on the system. AMBER99 forcefield was used in assigning correct electronic charges and protonation of amino acid residues. The 3D structure for rhodoquinol was built within MOE and energy minimized using the Amber10:EHT forcefield. A second minimization was applied using the MOPAC semi-empirical energy functions (PM3 Hamiltonian). Rhodoquinol was docked into the binding site of the AOX using the Triangle Matcher placement method with London dG scoring. Subsequently, poses resulting from the placement stage were further refined using the Induced Fit method, which allows protein flexibility upon ligand binding, improving the prediction accuracy for the interaction. Poses were then rescored using the GBVI/WSA dG scoring function and the top five best scoring poses were retained.

## Results

### AOX primary sequence analysis

The AOX EST cluster originally identified in the *Blastocystis* NandII strain EST data (Stechmann et al., 2008) appeared to be chimeric with a 40S ribosomal protein. Rapid amplification of cDNA ends (RACE) allowed the full 5′ and 3′ ends of the AOX gene to be obtained. The obtained sequence is identical to the one found in the recently completed *Blastocystis* sp. NandII genome (Gentekaki et al., 2017). The *Blastocystis* AOX gene encodes for a 304 amino acid protein with a predicted molecular weight of 35 kDa. The *Blastocystis* AOX sequence has been deposited into GenBank (accession number: FJ647192).

**Figure 1:**
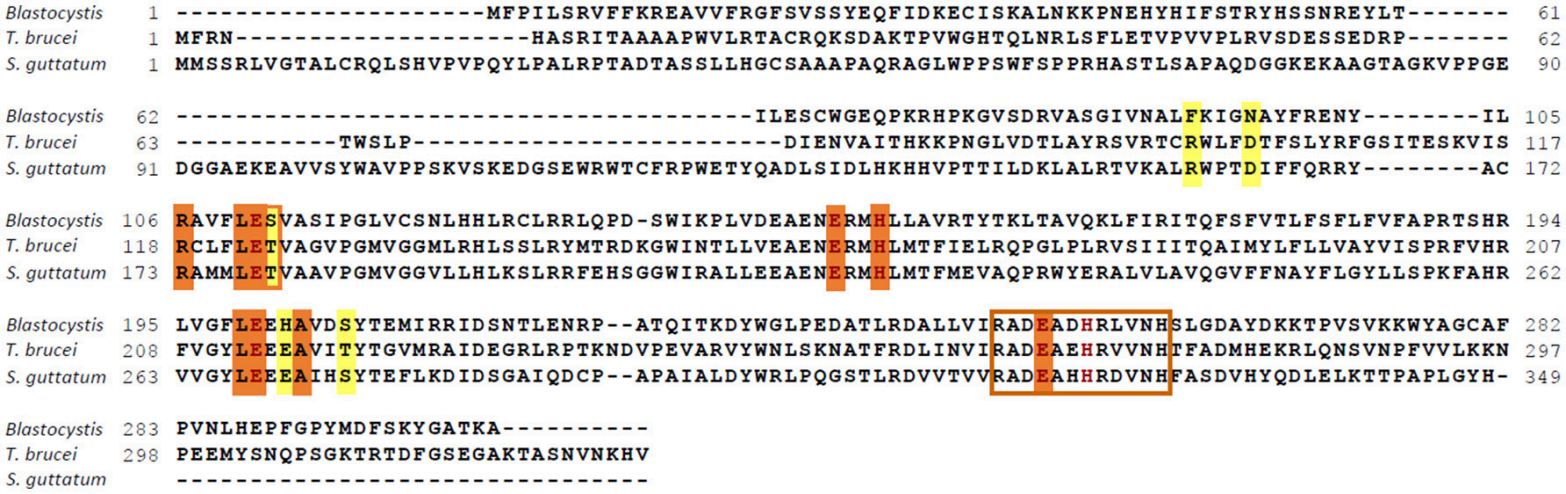
Functional residues are conserved in the *Blastocystis* alternative oxidase (AOX). The *Blastocystis* AOX was aligned to the *Trypanosoma brucei* and *Sauromatum guttatum* AOX sequences. Residues involved in coordinating the diiron in the active site are indicated in brown. Quinone binding residues are indicated by an orange background and possible rhodoquinol coordinating residues are indicated by a yellow background. The *S. guttatum* T179 postulated in oxygen affinity has been indicated by a yellow background and an orange rim. The epitope recognized by the *S. guttatum* AOX monoclonal antibody is indicated by a brown box.

**Figure 2:**
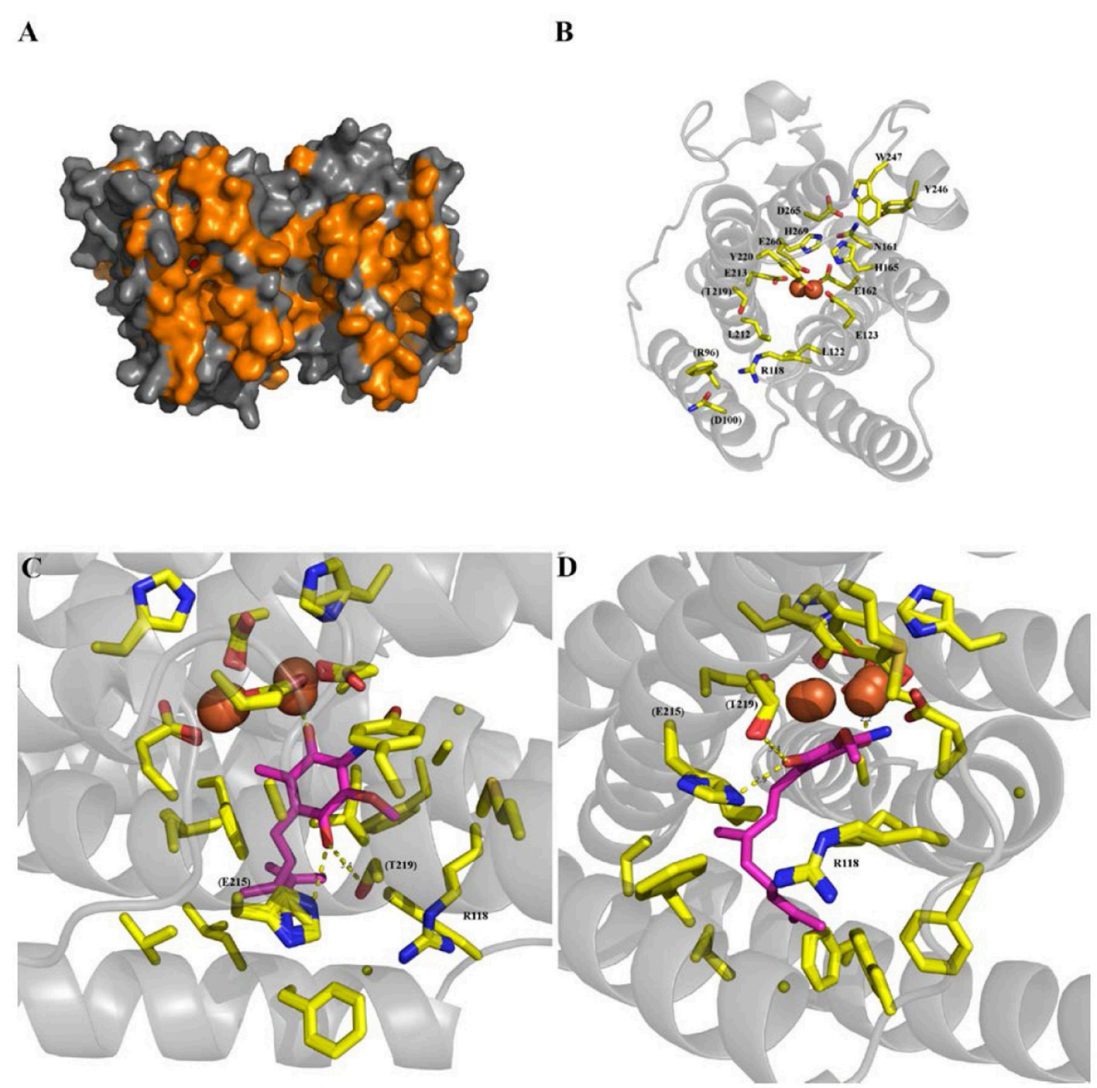
*Blastocystis* alternative oxidase (AOX) homology modeling from the trypanosomal alternative oxidase (TAO) crystal structure (PDB:5GN2) generated using the Swiss-model software (http://swissmodel.expasy.org/). **(A)** Surface representation of the model, with hydrophobic residues colored orange. **(B)** Primary and secondary ligation sphere, with numbering based on the TAO amino acid numbering. Non-conserved amino acids are labeled in parenthesise. **(C,D)** Show the same docked rhodoquinol (magenta) from two different orientations. Amino acids shown as sticks are all within 6 Å of the substrate, with potential hydrogen bonds to (E215H) (T219S) and the iron core shown as yellow dotted lines. Atoms are colored as yellow for carbon, blue for nitrogen and red for oxygen, with the iron core as orange spheres.

*Blastocystis* NandII, *Sauromatum guttatum* and *Trypanosoma brucei* AOX sequences were aligned to determine if residues known to be important for catalysis in other species were present in the *Blastocystis* homolog. The alignment shown in Figure 1 clearly demonstrates that many of the conserved features associated with AOX are present in the *Blastocystis* sequence. A surface model of the *Blastocystis* AOX, using the trypanosomal alternative oxidase (TAO) crystal structure as a template, is depicted in Figure 2A. The orange coloring indicates the hydrophobic surface and the membrane face depicted in Figure 2A is the surface which interacts with the mitochondrial inner membrane. Figure 2A also shows the hydrophobic cavity leading to the di-iron center. The residues lining the active site, which coordinate the diiron center (namely the 1° ligation sphere; *T. brucei* numbering throughout: E123, E162, E213, E266, H165 and H269), are all conserved (Shiba et al., 2013; Figure 2B). In addition, Figure 2B also illustrates that residues involved in the 2° ligation sphere (N161, Y220, D265, Y246, and W247), which function in electron transport and the oxygen reduction cycle are also conserved in the *Blastocystis* sequence (Affourtit et al., 2002; Moore and Albury, 2008; Young et al., 2016).

Interestingly, however, several of the residues involved in substrate and inhibitor-binding are different in *Blastocystis* compared to *T. brucei*. Although the majority of these residues are conserved (such as R118, L122, E123, A216, E162, H165, L212, E213, A216 and E266), several residues shown to interact with the tail of both substrate and inhibitors in *T. brucei* have been modified (as depicted in Figure 2B: R96F, D100N, and T219S). Since *Blastocystis* is an anaerobe it seems unlikely that it utilizes ubiquinone as substrate but probably uses rhodoquinol instead (Stairs et al., 2018) which operates at a much lower midpoint potential.

In order to assess the influence of these substitutions upon substrate binding, docking studies of rhodoquinol were undertaken using the homology model described in Figures 2C,D. As shown in Figure 2C, rhodoquinol is bound in a fashion analogous to the position determined for ubiquinol within the TAO crystal structure (Shiba et al., 2013), with the binding positions for 1-OH and 4-OH positioned between the iron core and T219S, respectively. What is apparent from Figures 2C,D is that the proposed proton transfer network within TAO (involving R96, D100, and E215; Young et al., 2016) is completely missing, and appears to have been replaced instead by a single histidine. Given the proximity of this histidine to the proposed rhodoquinol binding site, ~2.9 Å from the OH, and the likelihood it has free rotation around the R-group due to lack of a secondary binding point, it is highly likely that the histidine is able to act as a pathway for proton removal to solvent, thereby fulfilling a similar role within the quinol reactivity mechanism as the previously described pathway (Young et al., 2016).

As rhodoquinol is subtly different from ubiquinone, the residues which are different from *T. brucei* TAO might actually coordinate the rhodoquinol in the *Blastocystis* AOX. In agreement with this assertion is the discovery of *RquA* on the *Blastocystis* genome (Gentekaki et al., 2017; Stairs et al., 2018), a gene thought to encode an enzyme of the rhodoquinone biosynthetic pathway (Lonjers et al., 2012; Stairs et al., 2018). Similar to other parasites including microsporidia, *Blastocystis* does not contain any of the cysteine residues which, in plants at least, are thought to be involved in AOX activation by pyruvate (Rhoads et al., 1998). Its absence in *Blastocystis* suggests that this organism, similar to the microsporidian and the trypanosomal AOX, is not regulated by α-keto acids. In addition, T124 which has been linked to changes in oxygen affinity (Moore and Albury, 2008) has been changed to a serine residue in *Blastocystis*.

### *Blastocystis* AOX protein is mitochondrial

**Figure 3:**
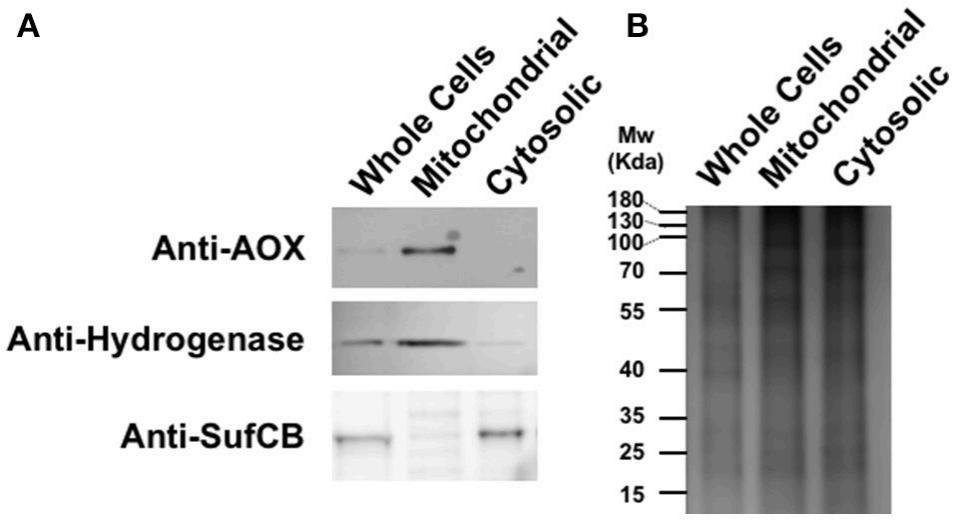
The *Blastocystis* alternative oxidase (AOX) is enriched in mitochondrial fractions. **(A)** Western blot analyses of the expression and cellular localization of *Blastocystis* AOX, the *Blastocystis* mitochondrial marker hydrogenase and cytosolic marker SufCB. **(B)** Typical SDS-PAGE gel of protein extracts from whole cells, mitochondrial and cytosolic fractions of *Blastocystis* stained with Coomassie blue.

Comparing the *Blastocystis* AOX sequence with the *Sauromatum guttatum* AOX indicates that the epitope for the *S. guttatum* AOX monoclonal antibody (RADEAHHRDVNH) is quite conserved in *Blastocystis* NandII. Of the twelve residues, ten are identical (see Figure 1). We therefore decided to test the *S. guttatum* antibody on *Blastocystis* NandII total protein extracts. Western blotting of *Blastocystis* fractions detected a single protein, which is enriched in the mitochondrial fraction, of ~29 kDa, in reasonable agreement with the predicted molecular weight for the *Blastocystis* AOX (Supplementary Figure 1A). Targeting signal predictions, such as Mitoprot (Claros and Vincens, 1996) and pSORT (Horton et al., 2007) failed to predict a mitochondrial targeting signal which could have explained the size difference between the observed and calculated molecular weight of the *Blastocystis* AOX. Amino acid composition and globularity of a protein do play a role in the actual observed molecular weight and membrane proteins are known the have issues in this regard (Rath et al., 2009). Using the anti-AOX antibody on *Blastocystis* cellular fractions clearly indicated an enrichment in mitochondria (Figure 3A). The AOX band appeared in the mitochondrial fraction, but absent in the cytosolic fraction, consistent with the absence of this protein in *Blastocystis* MROs. The anti-*Blastocystis* hydrogenase antiserum shows specific detection of *Blastocystis* hydrogenase (Stechmann et al., 2008) in the MRO fraction (MRO positive control) while the anti-*Blastocystis* SufCB antiserum shows specific detection of *Blastocystis* SufCB (Tsaousis et al., 2014) in the cytosolic fraction (positive control for cytosolic fraction).

**Figure 4:**
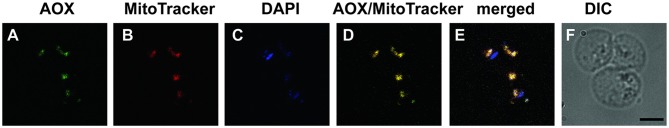
The *Blastocystis* alternative oxidase (AOX) is localized in the mitochondrion. Several *Blastocystis* cells are shown. **(A)** Anti-AOX antibody recognizes several discrete locations in *Blastocystis*. **(B)** Staining of the mitochondrion-like organelles with MitoTracker. **(C)** DAPI staining of DNA in the mitochondria and in the nucleus. **(D)** Overlay of anti-AOX and Mitotracker demonstrating the co-localization of signal. **(E)** Merged of all signals with co-localization of anti-AOX, Mitotracker and DAPI in the mitochondria and DAPI alone for the *Blastocystis* nuclei. **(F)** DIC image of the *Blastocystis* cells. Bar is 5 μm.

The *S. guttatum* AOX monoclonal antibody was subsequently used to localize the AOX within *Blastocystis* cells using immunofluorescence microscopy. The AOX antibody signal was found to co-localize with MitoTracker Red CMXRos (Figure 4), a mitochondrion-specific stain which has been used previously on *Blastocystis* mitochondria (Stechmann et al., 2008; Tsaousis et al., 2009, 2012). It also co-localized with the mitochondrial DAPI label in agreement with the presence of an organellar genome (Nasirudeen and Tan, 2004; Pérez-Brocal and Clark, 2008). This clearly suggests that AOX localized to the mitochondrion-related organelle found in *Blastocystis*.

### *Blastocystis* AOX complements heme deficient *E. coli*

To assay AOX activity, the *Blastocystis* AOX gene was expressed in a heme deficient *Escherichia coli* strain [FN102 (ΔhemA (Km^R^)) (Nihei et al., 2003)], where the gene for glutamyl-tRNA reductase, the first enzyme in heme biosynthesis, has been replaced with a kanamycin resistance gene. Expressing AOX in this strain complements for the heme deficiency as it provides *E. coli* with an additional terminal oxidase which does not require heme for activity (Fukai et al., 2003). Therefore, heme deficient cells expressing recombinant AOX do not require the addition of aminolevulinic acid, a heme precursor, which heme deficient cells normally require for aerobic growth (Fukai et al., 2003). In addition, expressing AOX in a heme deficient mutant reduces the potential for confusing AOX activity with the activity of other quinol oxidases. The main oxidases in *E. coli* use heme prosthetic groups for activity. *E. coli* FN102 cells capable of growth without aminolevulinic acid were further analyzed for the presence the *Blastocystis* AOX. This protein could indeed be detected in a purified membrane fraction from the heme deficient *E. coli* FN102 strain (Supplementary Figures 1B, 2).

### *Blastocystis* AOX uses oxygen and duroquinol and is sensitive to octyl gallate

**Figure 5:**
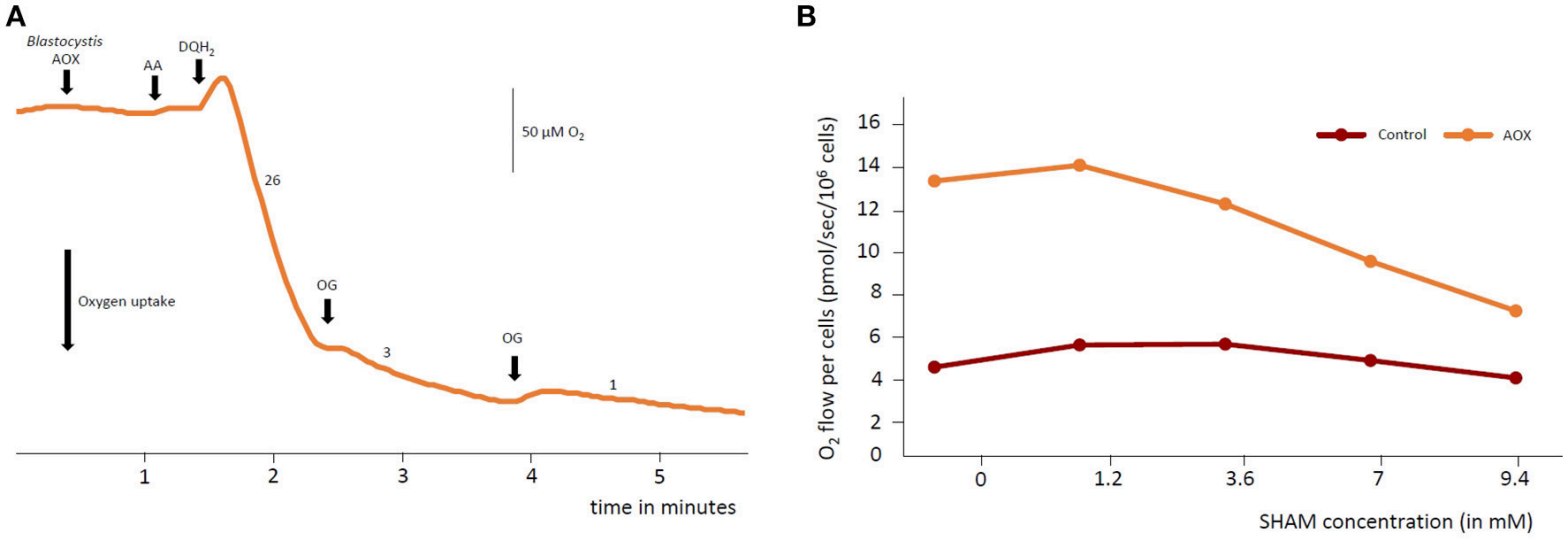
Oxygen uptake by *Blastocystis* alternative oxidase (AOX) in *Escherichia coli*. **(A)** Oxygen levels were allowed to stabilize before addition of heme deficient *E. coli* membranes expressing recombinant *Blastocystis* AOX. Addition of duroquinol (DQH2) (final concentration of 1 mM) induced oxygen consumption. Oxygen uptake was sensitive to a typical AOX inhibitor octylgallate (OG). Octylgallate was added to a final concentration of 25 μM. Oxygen consumption was not due to the action of complex IV as protein was expressed in heme deficient *E. coli* and functional complex IV cannot be produced by these cells. Furthermore, antimycin A, a complex III inhibitor, was added (AA) to a final concentration of 1 μM. Rates shown on the graph are nmols O_2_ consumed/min/mg protein. **(B)** Oxygen consumption by whole *E. coli* FN102 cells expressing *Blastocystis* AOX (orange trace) was measured using a high-resolution respirometer compared to *E. coli* cells not expressing the *Blastocystis* AOX (brown trace). Oxygen consumption is roughly three times higher in the AOX expressing strain and sensitive to the AOX inhibitor salicylhydroxamic acid (SHAM). Three independent experiments were conducted and a representative data set is presented.

The activity of AOX can be measured by oxygen uptake with quinols as substrates. Figure 5A shows the results of measuring oxygen uptake in the purified membranes of heme deficient *E. coli* expressing recombinant *Blastocystis* AOX. Addition of duroquinol (Sigma), an AOX substrate, initiates oxygen consumption indicating that oxygen uptake only occurs in the presence of quinols. Oxygen consumption is almost completely abated by the addition of octylgallate, an AOX inhibitor, clearly indicating that the oxygen consumption was due to the activity of an AOX. To demonstrate that the *Blastocystis* AOX is also sensitive to salicylhydroxamic acid (SHAM), another AOX inhibitor, we measured the oxygen consumption rate of the heme deficient *E. coli* FN102 strain expressing the *Blastocystis* AOX in whole cells. Similarly, oxygen consumption was affected by the AOX inhibitor (Figure 5B). Together, this data suggests that the *Blastocystis* AOX consumes oxygen in the presence of quinols and is sensitive to typical AOX inhibitors.

### *Blastocystis* cells respire molecular oxygen

**Figure 6:**
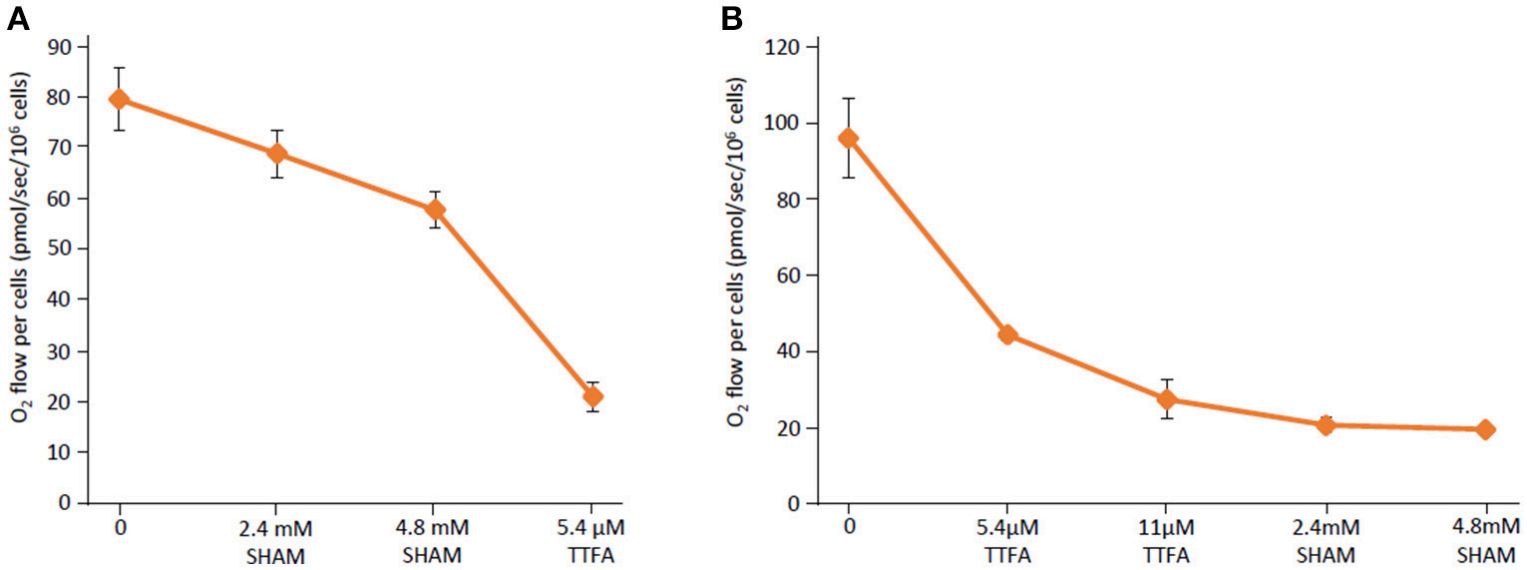
The *Blastocystis* alternative oxidase (AOX) is sensitive to salicylhydroxamic acid and thenoyltrifluoroacetone. **(A)** Routine respiration was determined in *Blastocystis* cells diluted to a density of 1 × 10^6^ cells/ml before the addition of the AOX inhibitor salicylhydroxamic acid (SHAM) in two sequential 2.4 mM doses. Following SHAM addition the succinate dehydrogenase (Complex II) inhibitor thenoyltrifluoroacetone (TTFA) was added to a final concentration of 5.4 μM. Shown are an average of four independent experiments. Error bars represent standard deviation. **(B)** Routine respiration was determined in *Blastocystis* cells diluted to a density of 1 × 10^6^ cells/ml before the addition of two doses of the Complex II inhibitor TTFA was added to a final concentration of 5.4 and 11 μM. Following this the AOX inhibitor SHAM was added in two doses to a final concentration of 2.4 and 4.8 mM. Shown are an average of two independent experiments. Error bars represent standard deviation.

In order to assess whether *Blastocystis* cells themselves are able to use molecular oxygen *in vivo*, whole *Blastocystis* cells were analyzed in a high-resolution respirometer. The oxygen consumption rate was measured in washed *Blastocystis* NandII cells at a density of 1 × 10^6^ cells/ml at 28°C. Live *Blastocystis* cells consumed oxygen and this activity was affected by addition of SHAM (Figure 6A). As AOX receives its electrons from Complex II (succinate dehydrogenase/fumarate reductase), we tested the effect of the Complex II inhibitor thenoyltrifluoroacetone (TTFA) on *Blastocystis* oxygen consumption. Similar to SHAM, exposure to TTFA also affects the oxygen consumption rate of *Blastocystis in vivo* (Figure 6B) suggesting the *Blastocystis* AOX does indeed receive its reducing equivalents via Complex II (see Supplementary Figure 3 for controls).

## Discussion

*Blastocystis* is the most common eukaryotic inhabitant of the human gut (Gentekaki et al., 2017). It has been marred by confusion almost from its first discovery in the nineteenth century when it was linked to cholera (see Zierdt, 1991). It has since then been associated with almost every eukaryotic domain until it was clearly shown to be a member of the large stramenopile lineage (Silberman et al., 1996). Stramenopiles are an extremely diverse grouping and can be found in many environments. It includes major plant pathogens, such as *Phytophthora*, but also diatoms which are major primary producers in the world's oceans. Together with *Pythium* (Hilton et al., 2016), *Blastocystis* is thought to be the only human pathogen in this eukaryotic lineage. However, reports about its supposed pathogenicity or role in disease are conflicting (see for example Miller and Minshew, 1988; Clark et al., 2013; Stensvold and van der Giezen, 2018). Presence of *Blastocystis* in stool samples of patients with gastrointestinal complaints has repeatedly been reported. However, as it a faecally-orally transmitted organism, people with *Blastocystis* in their intestines might also have been exposed to other potential pathogens and a causative relationship between disease and *Blastocystis* has never been demonstrated.

In the literature, *Blastocystis* is often associated with irritable bowel syndrome (IBS) although here again, the literature is conflicting. Several cohort studies suggest a link between *Blastocystis* and IBS (Yakoob et al., 2010; Jimenez-Gonzalez et al., 2012; Nourrisson et al., 2014) while others do not (Petersen et al., 2013; Krogsgaard et al., 2015; Beghini et al., 2017). A possible explanation for these disparate findings is the large genetic diversity observed within *Blastocystis* (Gentekaki et al., 2017) where some subtypes might indeed be linked to disease while others might not (Stensvold et al., 2007).

A factor that has thus far been overlooked in this respect is the fact that *Blastocystis* is considered to be a strict anaerobe (Zierdt, 1986) as it is incapable of oxidative phosphorylation (Fenchel and Finlay, 1995). Genomic studies confirm the notion that *Blastocystis* is indeed incapable of oxidative phosphorylation as the classic cytochrome *c* oxidase (Complex IV) is absent from its genome (Denoeud et al., 2011; Gentekaki et al., 2017). Indeed, only Complex I and II are present suggesting an anaerobic energy metabolism (Müller et al., 2012). The presence of an alternative oxidase in *Blastocystis* is therefore somewhat surprising as it suggests its energy metabolism might not be completely independent of molecular oxygen. Alternative oxidases are generally considered to be energetically wasteful enzymes as they normally short-circuit the mitochondrial electron transport chain by shuttling reducing equivalents from Complex I or II away from the proton translocating respiratory pathway to molecular oxygen without pumping protons across the mitochondrial inner membrane (Shiba et al., 2013; May et al., 2017). In plants AOX acts as a redox sink under stress conditions, such as through respiratory inhibition or drought thereby reducing the formation of deleterious reactive oxygen species (ROS) production.

It is interesting to speculate as to whether it plays a similar role in *Blastocystis* by not only providing a route of electron transport from Complex I and II, and in doing so generate a protonmotive force via Complex I, but also reduce ROS production at Complex I and through reversed electron transport from Complex II. Obviously, further experiments are required to substantiate such a notion. Nevertheless, the result that the Complex II inhibitor thenoyltrifluoroacetone inhibits alternative oxidase function in whole *Blastocystis* cells suggests that the alternative oxidase operates indeed via Complex II as in other organisms (Stechmann et al., 2008).

The oxygen concentration in a healthy gut is extremely low (Albenberg et al., 2014) to support growth of obligate anaerobic bacteria from the Firmicutes and Bacteroides phyla. These bacteria are important in maintaining a healthy gut ecosystem (Donaldson et al., 2016). When the microbial flora in the gut gets disturbed as in a dysbiotic gut, facultative anaerobic *Enterobacteriaceae* establish themselves resulting in an increase of the oxygen concentration (Rigottier-Gois, 2013; Byndloss et al., 2017; Rivera-Chávez et al., 2017). This suggests that for a strict anaerobe, such as *Blastocystis*, a dysbiotic gut is not the most ideal ecosystem and that similarly to obligate anaerobic bacteria, it no longer can maintain itself in this niche. Our data suggests that its alternative oxidase might allow it to deal with fluctuating oxygen concentrations that it might encounter in the gut. Similarly, it was previously shown that *Blastocystis* also has other mechanisms to deal with such oxygen fluctuations. In addition to the standard eukaryotic mitochondrial oxygen-sensitive iron-sulfur cluster assembly system, *Blastocystis* contains a SufCB protein that is expressed under oxygen-stressed conditions (Tsaousis et al., 2012). This might explain why some studies do not find *Blastocystis* in IBS patients (Petersen et al., 2013; Krogsgaard et al., 2015; Beghini et al., 2017) and others do (Yakoob et al., 2010; Jimenez-Gonzalez et al., 2012; Nourrisson et al., 2014). In well-established IBS guts, the dysbiosis might have driven *Blastocystis* out of the gut while in early stages of disease, the normal gut colonizer might attempt to stay by means of utilizing its alternative oxidase to protect itself from molecular oxygen as has been suggested earlier (Gomes et al., 2001; Stensvold and van der Giezen, 2018). Overall, our data suggests that *Blastocystis* can cope with fluctuating oxygen concentrations that it might encounter in the human gut and might be better described as a microaerophile. However, considering its overall oxygen-independent energy metabolism (Müller et al., 2012; Gentekaki et al., 2017), it seems unlikely that the dysbiotic gut of IBS patients is a suitable habitat for this anaerobe.

## Author contributions

AT, CG, LY, AM, and MvdG designed the study. AT, KH, CE, LY, AR-H, and CG performed experiments. All authors analyzed the data. MvdG and AM wrote the first draft and all authors reviewed and edited the final text.

### Conflict of interest statement

The authors declare that the research was conducted in the absence of any commercial or financial relationships that could be construed as a potential conflict of interest.

## Abbreviations

AOX: alternative oxidase
DDT: dichloro diphenyl trichloroethane
EST: expressed sequence tag
IBS: irritable bowel syndrome
ROS: reactive oxygen species
SHAM: salicylhydroxamic acid
TAO: trypanosomal alternative oxidase
TTFA: Thenoyltrifluoroacetone
