# Supplementary material for: The Human Gut Colonizer Blastocystis Respires Using Complex II and Alternative Oxidase to Buffer Transient Oxygen Fluctuations in the Gut
Source: Front Cell Infect Microbiol. 2018 Oct 22;8:371. doi: 10.3389/fcimb.2018.00371 (PMC6204527; doi:10.3389/fcimb.2018.00371)
Supplement: Supplementary file 1 [file Data_Sheet_1.PDF]

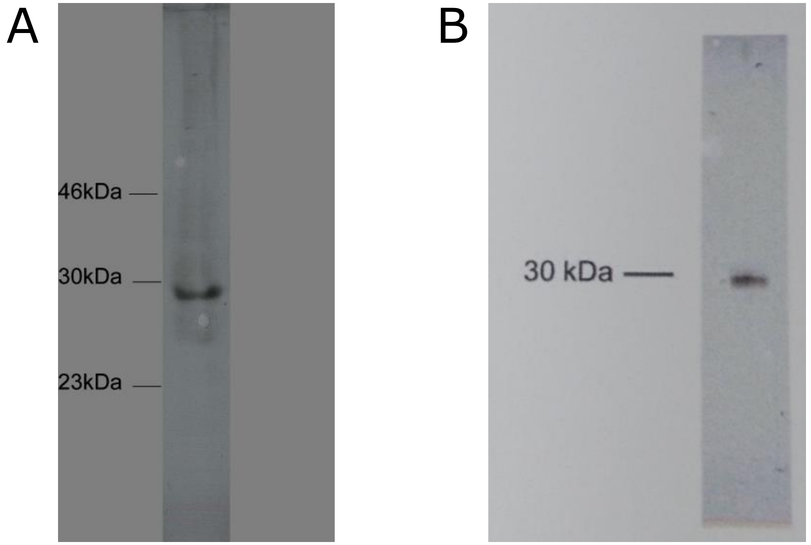

Supplementary Figure 1. The *Sauromatum guttatum* (voodoo lilly) monoclonal antibody recognises the *Blastocystis* alternative oxidase (AOX). A. Immunoblot of *Blastocystis* total protein using the *S. guttatum* anti-AOX antibody recognises a single protein of approximately 29 kDa. B. Immunoblot of *Escherichia coli* FN102 expressing the *Blastocystis* AOX gene decorated with the *S. guttatum* anti-AOX antibody also recognises a single protein of 29 kDa.

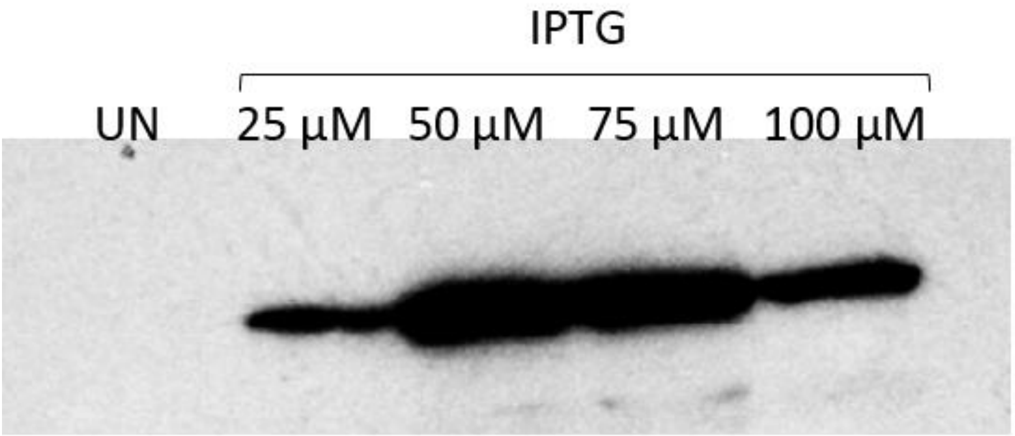

Supplementary Figure 2. Western blot analysis of *Escherichia coli* FN102 strain expressing *Blastocystis* alternative oxidase (AOX) using increasing IPTG concentrations, with UN indicating the un-induced lane. Recombinant protein detected using an anti-His tag antibody.

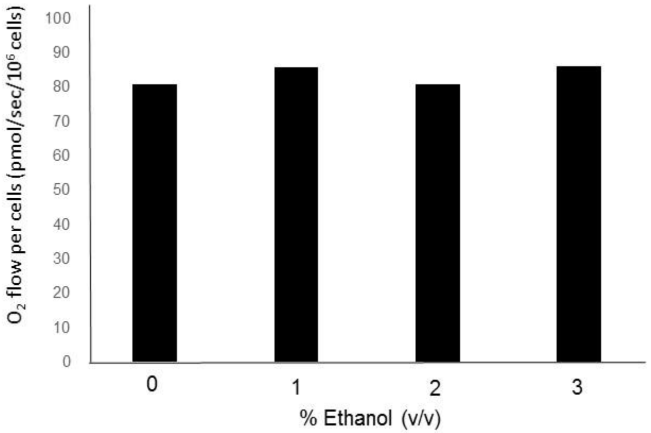

Supplementary Figure 3. *Blastocystis* respiration with addition of ethanol as solvent control for TTFA and SHAM addition experiments.
